# Biomechanical and Histological Analysis of Titanium (Machined and Treated Surface) Versus Zirconia Implant Materials: An In Vivo Animal Study

**DOI:** 10.3390/ma12060856

**Published:** 2019-03-14

**Authors:** Sergio Alexandre Gehrke, Juan Carlos Prados-Frutos, María Prados-Privado, José Luis Calvo-Guirado, Jaime Aramburú Júnior, Leticia Pérez-Díaz, Patricia Mazón, Juan Manuel Aragoneses, Piedad N. De Aza

**Affiliations:** 1 Department of Research, Biotecnos, Cuareim 1483, Montevideo CP 11100, Uruguay; 2 Department of Oral and Implant Surgery, Faculty of Health Sciences, Universidad Católica de Murcia (UCAM), 30107 Murcia, Spain; jlcalvo@ucam.edu; 3 Instituto de Bioingenieria, Universidad Miguel Hernández, Avda. Ferrocarril s/n, 03202 Elche (Alicante), Spain; pmazon@umh.es (P.M.); piedad@umh.es (P.N.D.A.); 4 Department of Medicine and Surgery, Faculty of Health Sciences, Rey Juan Carlos University, 28922 Madrid, Spain; juancarlos.prados@urjc.es; 5 Department of Continuum Mechanics and Structural Analysis, Carlos III University, 28911 Madrid, Spain; mprados@ing.uc3m.es; 6 Department of Surgery, Faculty of Veterinary, Faculty of Itapiranga, Itapiranga CP 89896000, Brazil; jaimearamburujunior@gmail.com; 7 Laboratorio de Interacciones Molecular, Facultad de Ciencias, Universidad de la Republica, Calle Iguá 4225, Montevideo 11400, Uruguay; letperez@gmail.com; 8 Department of Dental Research, Universidad Federico Henriquez y Carvajal (UFHEC), Santo Domingo 10107, Dominican Republic; jaragoneses@ufhec.edu.do

**Keywords:** osseointegration, bone healing, bone quality, zirconia implants, titanium implants

## Abstract

Objectives: The aim of this study was to perform an in vivo histological comparative evaluation of bone formation around titanium (machined and treated surface) and zirconia implants. For the present study were used 50 commercially pure titanium implants grade IV, being that 25 implants with a machined surface (TiM group), 25 implants with a treated surface (TiT group) and, 25 implants were manufactured in pure zirconia (Zr group). The implants (*n* = 20 per group) were installed in the tibia of 10 rabbits. The implants distribution was randomized (*n* = 3 implants per tibia). Five implants of each group were analyzed by scanning electron microscopy and an optical laser profilometer for surface roughness characterization. Six weeks after the implantation, 10 implants for each group were removed in counter-torque for analysis of maximum torque value. The remaining samples were processed, included in historesin and cut to obtain non-decalcified slides for histomorphological analyses and histomorphometric measurement of the percentage of bone-implant contact (BIC%). Comparisons were made between the groups using a 5% level of significance (*p* < 0.05) to assess statistical differences. The results of removal torque values (mean ± standard deviation) showed for the TiM group 15.9 ± 4.18 N cm, for TiT group 27.9 ± 5.15 N cm and for Zr group 11.5 ± 2.92 N cm, with significant statistical difference between the groups (*p* < 0.0001). However, the BIC% presented similar values for all groups (35.4 ± 4.54 for TiM group, 37.8 ± 4.84 for TiT group and 34.0 ± 6.82 for Zr group), with no statistical differences (*p* = 0.2171). Within the limitations of the present study, the findings suggest that the quality of the new bone tissue formed around the titanium implants present a superior density (maturation) in comparison to the zirconia implants.

## 1. Introduction

Titanium implants have become a common practice for replacing missing teeth. Although zirconia implants are gaining ground in clinical practice. They are not yet a clinical routine due to the lack of scientific and mechanical studies [1,2], although in the last few years, zirconia implants have been studied in detail as alternative biomaterials for replacement of missing teeth [3].

A large percentage of zirconium implants are formed by a tetragonal zirconium polycrystalline 3 molar (3Y-TZP). It has been demonstrated that this configuration does not cause an inflammatory reaction, no adhesion of proteins, adherence of cells oteoblásticas, cell adhesion, cell differentiation binding occurs implant and especially titanium [2].

Titanium and zirconia differ in many aspects and have their own advantages and disadvantages. Zirconia (ZrO_2_) is a polycrystalline ceramic dioxide of the transition metal zirconium (Zr) [4]. The advantages of zirconia are its low modulus of elasticity and thermal conductivity, low affinity to plaque, low corrosion and high biocompatibility [5], in addition to its white color. Many studies have found zirconia Young’s modulus between 200 and 210 GPa [6]. Zirconia has also less bacterial adhesion in the surface than titanium, therefore, biologic complications should be reduced [7]. However, the main disadvantage of zirconia implants is the low-temperature degradation (ageing) which results in degradation of the mechanical properties (strength, toughness and density of the material) [8,9]. Another important factor is that zirconia is more brittle and more vulnerable by bending and crack growth [10], so fracture resistance of zirconia dental implants is worse than titanium implants [11]. Due to its advantages, zirconia is becoming a material of great interest for dentistry, particularly where aesthetics are required [3].

Several in vitro studies have investigated the biocompatibility of zirconia and its osseointegration with the conclusion that it promotes proliferation of osteoblasts at levels greater than aluminum oxide [4,12]. Other studies have shown that zirconia implants reduce bacterial colonization, and therefore, the risk of periimplantitis [13]. Biocompatibility has been also proven in several animal investigations [14,15]. Osseointegration of zirconia implants has been demonstrated in several in vivo experiments where the conclusion was that that osseointegration is comparable to the level achieved with titanium alloys [16,17].

Ceramic materials, as zirconia, have an important sensitivity to surface defects which may generate cracks. These cracks can penalty the mechanical properties of zirconia dental implants [18]. Due to the mechanical properties of titanium and zirconia are different, usual geometries employed in titanium implants cannot be transferred to zirconia designs [19]. Zirconia is sensitive to subcritical crack growth and bending, so sharp edges, as common in titanium implants, should be avoided [19,20]. For this reason, most zirconia dental implants are one-piece or two-piece systems with a bonded abutment [5].

The main inconvenience of employing a two-piece implant with the abutment bonded to the implant is that, in case of failure, the entire implant must be removed. Some screwed implant-abutment connections have been developed with the aim of reducing these limitations [5,21]. Some in vitro studies have concluded that the geometry of implant-abutment connection has a crucial influence of zirconia abutments behavior [22]. Failures in two-piece systems always involve the connecting screw because zirconia does not tolerate tensile forces, which appear around the screw [23]. In addition, zirconia is sensitive to ageing in the presence of water, which is an oral environment is crucial because this ceramic will become a brittle behavior [1].

There is an interest in the use of zirconia in dental implants due to the increase in the number of published studies in the last few years on this topic [24]. However, limited in vivo and in vitro research data are available regarding the performance of zirconia for dental applications.

Thus, the aim of this study was to compare the performance of aspects related to osseointegration (biomechanics and histologic aspects) of titanium (machined and treated surface) versus zirconia implants inserted in tibias of rabbits after a period of 6 weeks.

## 2. Materials and Methods

Animals and experimental groups: Ten New Zealand adult female rabbits with a mean weight of 4.0 kg, were used in this study. The experiment was performed in accordance with the Brazilian guidelines and regulations, i.e., followed the standards of animal welfare in accordance to the Sociedade Brasileira de Ciência de Animal de Laboratório”, SBCal (http://www.cobea.org.br) and the Brazilian federal law regulating the issues related to animal research that was published in October 2008 (http://www.planalto.gov.br/ccivil_03/_Ato2007-2010/2008/Lei/L11794.htm). The study was approved by the ethics committee of the Veterinary Medicine of the Faculty of Itapiranga (Itapiranga, Brazil-#004-09-2015), and the animals received all the care stipulated by the institution.

**Figure 1:**
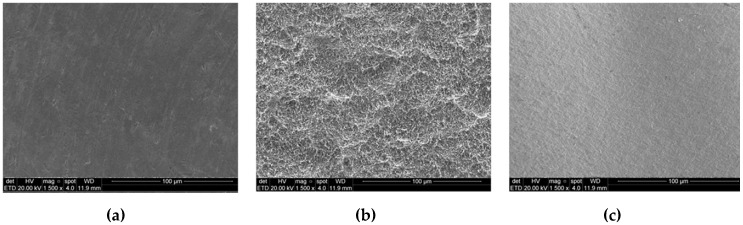
Scanning Electronic Microscopy images of the implant surface of (**a**): TiM group, (**b**): TiT group and (**c**): Zr group, respectively. The increase of 1.500×.

Sixty special mini-implants with 2.2 mm in diameter and 4 mm in length were manufactured specially for this study by Implacil De Bortoli Company (São Paulo, Brazil) in two different materials. The implants were divided into three groups (*n* = 25 per group): two titanium groups, where the implants manufactured in commercially pure titanium grade IV, which 25 implants were machined (TiM group) and a smooth surface was obtained (Figure 1a), and 25 implants were the surface was treated (TiT group) with sandblasted acid-etched using TiO_2_ particles with 100 µm to blasting and maleic acid to the conditioning (Figure 1b); Zr group, where the implants were produced in yttrium-stabilized tetragonal zirconia polycrystal (Y-TZP), which was standardized from CAD-CAM blocks (Figure 1c). Then, the specimens were treated, sterilized and packed using the same protocol standardized by the implants commercialized in the market.

Implants characterization: A profiler software (Leica DCM 3D Dual Core, version for Windows, Leica Microsystems Ltd., Heerbrugg, Switzerland) calculated the surface roughness parameters *S*_a_ and *R*_a_. *S*_a_ measurement was performed in the total area (254.64 × 190.90 µm^2^) and *R*_a_ was measured in a length of 254.64 µm. The mean of roughness values of *S*_a_ and *R*_a_ and standard deviation were calculated from the five profiles of each specimen group. The meaning of the surface roughness parameters *S*_a_ and *R*_a_.

**Figure 2:**
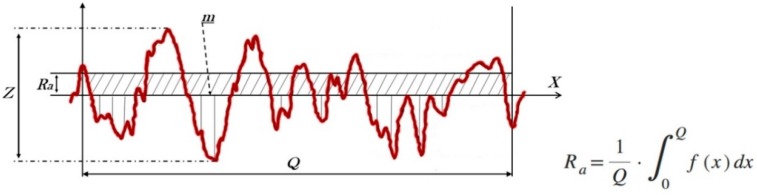
Representative scheme of the parameters measurement (*S*_a_, *R*_a_ and *Z*) on the surface samples and the arithmetic average roughness calculation of the *R*_a_.

A roughness value can either be calculated on a profile (line) or on a surface (area). *R*_a_ is the parameter of the profile roughness parameter, which is the most employed, and *S*_a_ is a measure of area roughness. *R*_a_ means the value obtained by the following formula and expressed in micrometer (µm) when sampling only the reference length from the roughness curve in the direction of the mean line, taking *x*-axis in the direction of mean line and *y*-axis in the direction of longitudinal magnification of this sampled part and the roughness curve is expressed by *y* = *f*(*x*) (Figure 2):

*S*_a_ is the extension of *R*_a_ (arithmetical mean height of a line) to a surface. It expresses, as an absolute value, the difference in height of each point compared to the arithmetical mean of the surface. This parameter is used generally to evaluate surface roughness. Moreover, the longest distance recorded among the peak and valley, high variation of the valleys (*Z* parameter) was analyzed.

After the surface analysis, they were coated with a gold sputter (SCD 050; Bal-Tec RG, Balzers, Liechtenstein, Germany) and the surface morphology was observed on SEM (XL30 FEG; Philips, Eindhoven, The Netherlands) with the magnification of 1500×.

Surgical procedure: Initially the animals were pre-anesthetized intramuscularly with a dose of acepromazine maleate (0.2 mg/kg) and morphine sulfate (2 mg/kg and, then, ketamine chloride (10 mg/kg) and 1 mg midazolam (1 mg/kg) were administered intravenously under general anesthesia. Additionally, 1 mL of local anesthetic (3% Prilocaine-felypressin, Astra, Mexico) was subcutaneously injected at the site of surgery to improve analgesia and control bleeding. The trichotomy in both tibias and antisepsis with topical iodopovidone were performed.

**Figure 3:**
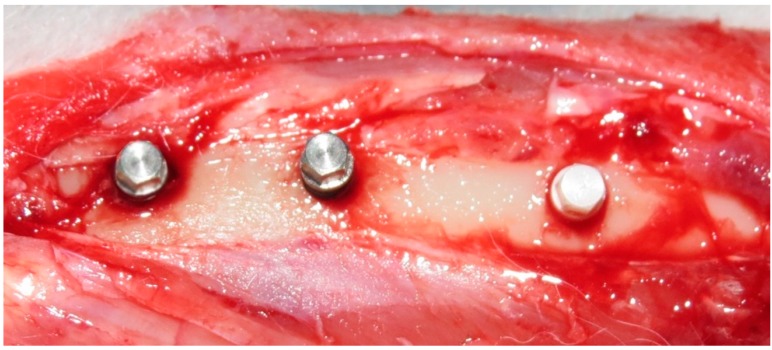
Representative image of the implant samples installed in an animal bone tibia.

The incision was 10 mm below the articulation in the skin and posteriorly in the fascia in the proximal-distal direction. Three perforations were made using a pilot spade drill with 2 mm in diameter and 5 mm in length with copious irrigation using saline solution. A distance of 10 mm between the three perforations was maintained. Then, the implants were manually installed at the bone level, with the hexagonal portion of the implant head out of the bone (Figure 3), controlled by an experienced surgeon (SAG). The animals were divided into 2 groups of 5 animals, for biomechanical test and histological analysis. Then, the implants were distributed by a randomized protocol (www.randomization.com) inside of the two lots (*n* = 3 implants per tibia). The suture was performed in two planes (muscular and subcutaneous) using a simple point, with nylon 4–0 (Johnson & Johnson/Ethicon, New Brunswick, NJ, USA).

A single dose of 600,000 IU Benzetacil (Eurofarma, São Paulo, Brazil) was used in animals related to the weight of animals. After the surgeries, the animals were housed in their own cages, with special care from a veterinarian, with diet ad libitum, soft glucose-free and kept at a temperature of 21 °C inside the cage. Six weeks after the implantations, all animals were sacrificed through an intravenous overdose of ketamine 2 mL (Agener Pharmaceutica, São Paulo, Brazil) and xylazine 1 mL (Bayer, São Paulo, Brazil). The tibias were removed and placed in 10% formalin solution and kept for one week for fixation.

Removal torque test: Both tibias of the lot of five animals, previously designed for the biomechanics test, were removed and processed immediately after the euthanasia for the measurement of the maximum removal torque of each implant to conserve the mechanical proprieties of the bone [25]. A similar procedure compared to other studies was performed by our group [26]. The removal torque test was performed using a computerized torque machine (CME, Técnica Industrial Oswaldo Filizola, São Paulo, Brazil), and the mean of maximum removal torque value was calculated for each group.

Histomorphological and histomorphometric procedures: The tibia bone blocks of another five animals containing the implants were dehydrated gradually in successive concentrations of alcohol (50%–100%) and embedded in glycol methacrylate resin (Technovit 9100 VLC, Kulzer, Germany) to produce the slice sections, which cut and ground sections that contained the central part of each implant and had a final thickness of 30 µm were produced using a macro cutting and grinding system (Isomet 2000, Buehler, Germany). Then, the sections were stained with picrosirius hematoxylin, and histomorphometric analysis was carried out. Finally, the sample was stained with picrosirius hematoxylin and analyzed under an optical microscope (Nikon Eclipse E200, Nikon Corporation, Tokyo, Japan).

**Figure 4:**
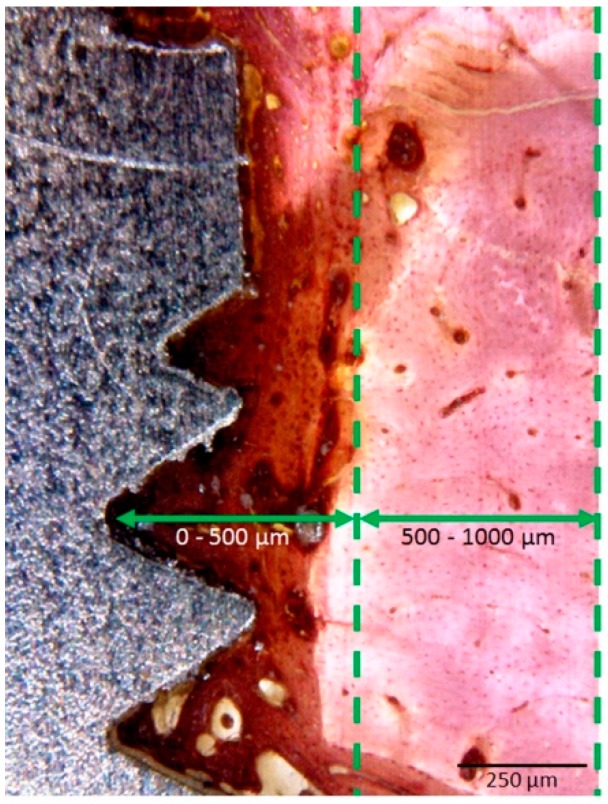
Histological section of the implant in the cortical bone portion (magnification 40×) showing 2 zones determined for histomorphometric analysis, which the first zone (0–500 µm) and the second zone (500–1000 µm).

The histomorphologic analysis was performed around all implants in order to establish the descriptive characteristics of the new bone present after the bone healing (osseointegration). The histomorphometric measurement of bone to implant contact percentage (BIC%) was performed at images with 50–200 times magnification using specific software (Image*J* for Windows, version 6, Research Services Branch, National Institute of Mental Health, Bethesda, MD, USA). The BIC% was calculated after the measurements of the points with the direct bone to implant contact around the implant and subtracted from the total implant perimeter. Moreover, another quantitative parameter measured was the bone volume percentage (BV%), which was determined using the methodology described in other studies [27,28], where the bone around the implant was divided into two zones: the first zone 1 (0–500 µm) and the second zone 2 (500–1000 µm). Figure 4 shows the two zones analyzed.

Statistical analysis: The ANOVA One-Way test was used to verify statistical differences among the groups. The comparison between the three groups in the same test was performed using the Mann-Whitney U-test. These statistical analyses were performed using the software GraphPad Prism 5.01 (GraphPad Software Inc., San Diego, CA, USA). The level of significance was set at α = 0.05.

## 3. Results

### 3.1. Surface Characterization Analysis 

SEM images showed a different surface morphology between the titanium machined surface, titanium treated surface and zirconia implants surface (Figure 1a–c). Table 1 shows the data of roughness parameters (*S*_a_, *R*_a_ and *Z*) of the groups. A highly significant difference in the surface roughness for the TiT group in compare to the TiM and Zr groups for all parameters (*p* < 0.0001).

### 3.2. Removal Torque Test

**Figure 5:**
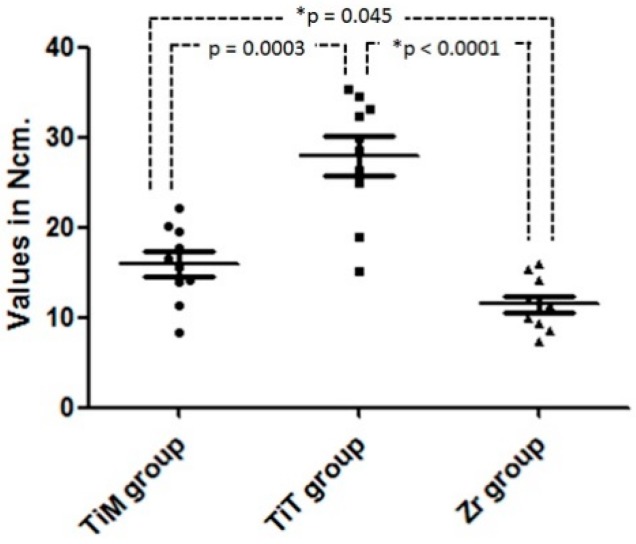
Graph of the removal torque dispersion values and statistical comparison of the groups. * Statistically difference (*p* < 0.05).

Table 2 shows the maximum removal torque values measured in the Ti and Zr groups. The values in the Zr group were, on average, half those in the Ti group (*p* < 0.0001). All values measured are presented with the dispersion and median in the graph of Figure 5.

### 3.3. Histomorphological Analysis

**Figure 6:**
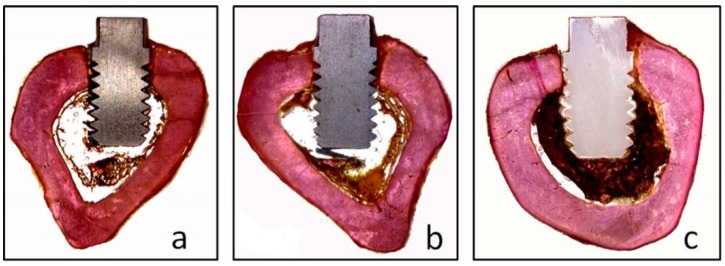
Images of the implants installed in the tibia of the three groups, (**a**) TiM group, (**b**) TiT and (**c**) Zr group.

Qualitative evaluation of the histological slides demonstrated that the most cervical portion of all implants passed through the tibial cortical bone, and the apical portion was in contact with medullary bone (Figure 6).

**Figure 7:**
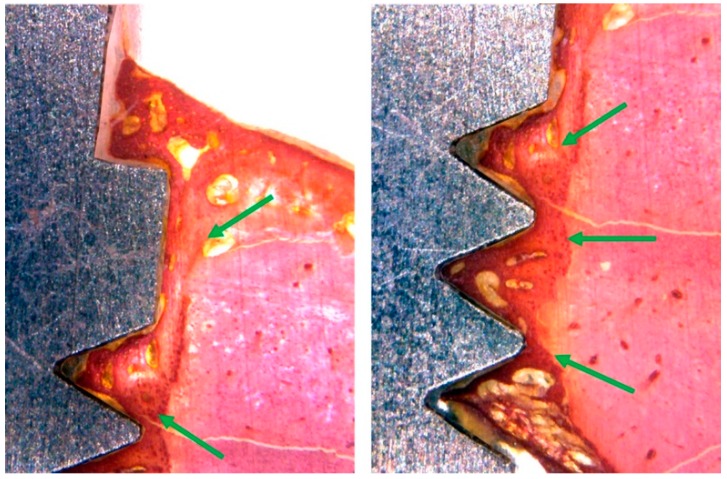
Representative histological images of the TiM group shows the bone to implant contact and a great quantity of bone matrix formation (red staining) around the surface (green arrows).

In the TiM group, a new bone formation was founded in different areas close to the implant surface, with regions of bone remodeling, similar to lamellar bone close to the implant, a large number of voluminous osteocytes was observed located within wide gaps. Moreover, immature bone trabeculae with large remodeling areas were observed. The difference in color staining (more intensely stained areas) showed more new formed bone, founded particularly in between the implant threads (Figure 7).

**Figure 8:**
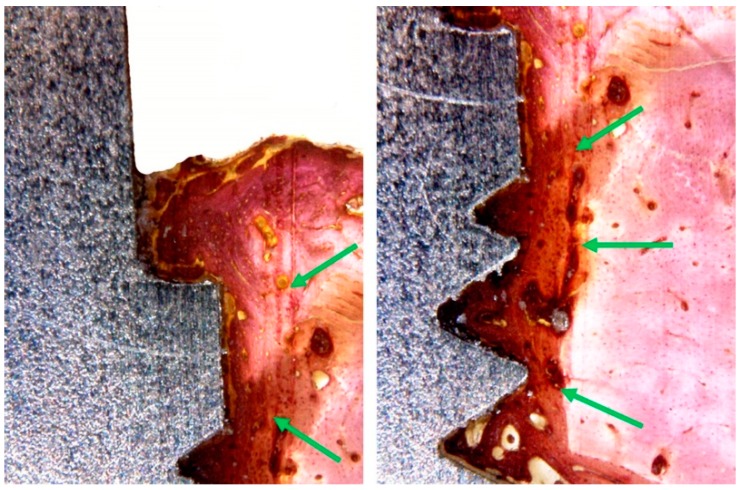
Histological images of the TiT group shows the bone to implant contact and a great quantity of bone matrix formation (red staining) around the surface (green arrows).

Whereas, in the TiT group, similar to the TiM group, the histological analysis showed bone neoformation in the areas adjacent to the implant surfaces, with regions of bone remodeling, showing evidence of a structural arrangement similar to that of the lamellar region. Around the implants, a superior bone density was observed and a minimum gap in the interface between bone and implant, with a smaller amount of collagen matrix present in these areas (Figure 8).

**Figure 9:**
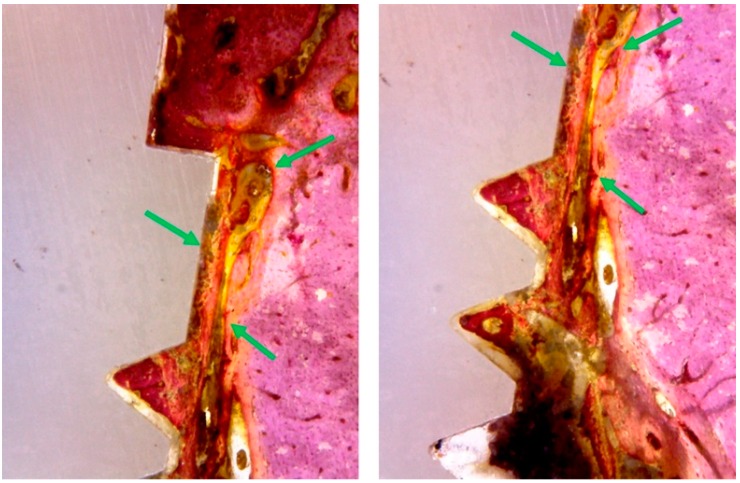
Histological images of the Zr group shows the bone to implant contact and the presence of collagen fibers and less intense density of the bone tissue around the surface (green arrows).

However, in the histological analysis of Zr group samples, showed a presence of bone neoformation in the areas adjacent to the implant surfaces, with sites of bone remodeling close to the tops of the spirals and in the more cervical portion of the implant. In some samples, extensive areas of collagen non-mineralized tissue were observed in contact with the implant surface (Figure 9).

### 3.4. Histomorphometric Analysis

**Figure 10:**
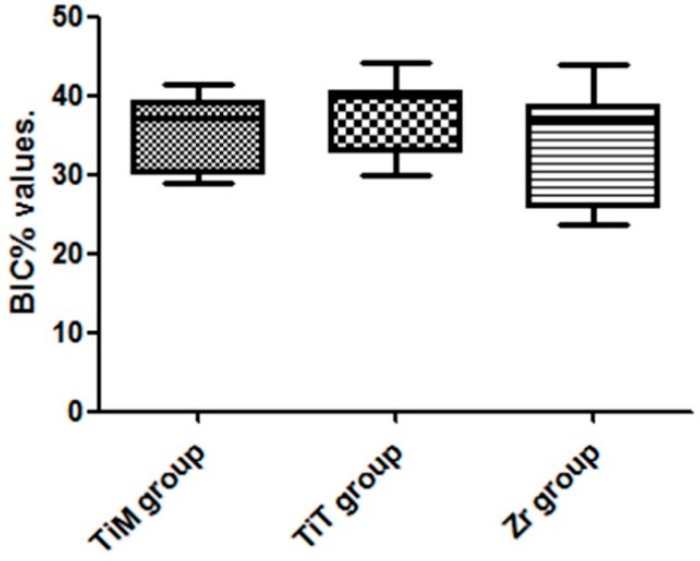
Box-Plot graph of the BIC% values of both groups.

The mean BIC% values in the Ti group were similar to the values of Zr group, showing no statistically significant difference among the groups (Table 3). The distribution of the BIC% values measured is presented in the box plots graph of Figure 10.

The groups showed significant differences in BV% (*p* = 0.0012) for the first zone evaluated (0–500 µm): 51% ± 6% for TiM group, 77% ± 5% for TiT group and 42% ± 5% for Zr group. In the second zone analyzed (500–1000 µm) all samples of the three groups showed a similar BV% with a mean superior to 90%, with no statistical differences (*p* > 0.05).

## 4. Discussion

The goal of this study was to compare the performance of aspects related to osseointegration of titanium (treated and not) and zirconia implants inserted in tibias of rabbits after a period of 6 weeks. This study analyzed the surface roughness (five implants per group), tested the biomechanical proprieties through the resistance to removal torque (10 implants per group) and, finally, measured the percentage of bone-implant contact and bone volume in two determinate areas (0 to 500 µm and 500 to 1000 µm) by a histomorphological analysis (10 implants per group). The results showed an important difference in the biomechanical test (torque removal) and in the bone volume at the measured area from 0 to 500 µm, but no statistical difference in the bone-implant-contact.

The physic-chemical composition and topography of the material surface implanted in the bone are directly related with the response of this tissue and, consequently, with the characteristics of the new tissue formation around of the material surface [28,29]. The present study showed a strong dense bone tissue response to titanium implants in comparison to zirconia implants after 6 weeks of healing in rabbit bone. Furthermore, the treated surface of the titanium implants showed resistance to removal torque forces superior to smooth surface and zirconia implants. These results can have a direct relationship with the fact related in other studies that showed the lower capacity of adhesion of osteoblasts cells on the zirconia structures in comparison with titanium structures [30].

The measurement of the percentage of bone to implant contact (BIC%) around the implants is considered as a parameter to evaluate the potential of osseointegration and it has been used to compare different implants with different macro- and micro-designs, materials or surface modifications [31,32]. The data obtained in our measurements, with respect to BIC%, showed very similar values between the groups, without statistical differences between the three groups, similar with other studies performed on animals comparing zirconia implants with titanium implants [33]. In this case, when we use only this parameter to determine the osseointegration of a material, it can be affirmed that zirconia is a good material to be used as an implant.

Another important parameter to evaluate the osseointegration is the test of torque removal of implants after different times of waiting for healing. In this way, the higher value on the removal torque can be interpreted as an increase in the bone-implant contact [34]. However, the measured value of the torque to remove the implant is directly related by the bone density (maturation and mineralization). This fact was described by Tabassum et al. (2014), showing that the implants evaluated in a period of 3 weeks after their implantation, even presenting high BIC% values, did not show great implant stability. This fact led the authors to propose the probability that this occurred due to the low calcification of the new bone formed at the interface with the implants [28]. During osseointegration of the implants, one of the phases is the formation of the bone matrix, which can be observed histologically and considered as “new bone” formed. However, in order for this scar tissue to have the necessary mechanical strength to withstand functional needs, proper mineralization is required, which will determine the strength (strength) of the tissue. Thus, through the difference presented between the histological and biomechanical results, we can deduce that titanium implants produce a much superior stimulus for bone matrix mineralization compared to zirconia implants. Correlating with a clinical scenario, early loads should be avoided on zirconia implants.

Removal torque is a common test for in vivo analysis to measure the quality of the bone in contact with the implant surface (osseointegration). Therefore, with the removal torque, it is possible to evaluate the strength of the interaction between the bone and implant surface [35]. Good osseointegration is characterized by high values of removal torques [34]. In view of the results obtained in this study, titanium implants obtained a better osteointegration than zirconia implants after 6 weeks. These results were highly significant, and it is thus concluded that there is an important effect among the groups. This is in accordance with other studies [35]. Results detailed in Table 1 show a bigger average in the removal torque values of 38.3% for the TiM group and 142.6% for the TiT group in comparison with the zirconia implants. Similarly, in the study presented by Gahlert et al. which compared the removal torque values of zirconia implants with titanium implants treated by sandblasted acid technique, the machined zirconia implants showed statistically significant lower values than the titanium implant after 8 weeks, being that the treated titanium implant showing values approximately four times bigger than the machined zirconia implants [36].

The time defeminated at 6 weeks in our study was based on previous studies reported in the revised literature [37,38,39], which concluded that the remodeling process of the bone-to-implant interface is complete in this time for the rabbit animal model. However, Halldin and coworkers demonstrated that the osseointegration of the implants is directly related to the type of bone, that is, it is distinct in trabecular bone than in cortical bone [40,41]. For this reason, and because of the size of the implants prepared for this experiment, the tibia model was selected where the implants, due to their size, were inserted in cortical bone almost half of their total length.

In accordance with Davies [42], the evaluation of the bone healing around the implant surface can be considered in two directions: (1) contact ossification, where the new bone formed evaluated is in direct contact to the implant surface after the cellular events, which was discussed previously as the bone to implant contact (BIC%); (2) ossification in distance, where the process of bone healing is evaluated since of the implant surface to the native bone of the implanted area, in this study called as bone volume (BV%). To determine the bone volume, in the peri-implant area was performed two lines equidistant (since of the implant to the native bone), in accordance to previous studies [27,28]: zone 1 (0–500 µm) representing the area of contact osteogenesis and, zone 2 (500–1000 µm) representing the transition zone for the native bone. Histomorphometric measurements of the samples revealed that for all three groups, the BV% was smaller in zone 1 as compared to zone 2. This can be explained by areas of bone neoformation where we found several zones with the presence of the collagen matrix. In this sense, the TiT group showed the bigger values of BV% in comparison with the TiM and Zr groups. These results are similar to Scarano et al., that demonstrated in their study that zirconia implants can form a great quantity of newly formed bone as the titanium implants [43]. This means that zirconia implants are highly biocompatible and osteoconductive. However, Hoffman et al. However, demonstrated that the zirconia implants obtained a similar rate of bone formation on zirconia and modified titanium surface with a high amount of bone apposition in all implants at 2 and 4 weeks in New Zealand white rabbits [44].

Moreover, when we evaluated the percentages of the neoformed bone area in the zone 1 and compared quantitatively, the results showed a present bone volume of 21.4% for the TiM group and 83.3% for the TiT group in relation to the Zr group. The highest percentage differences were found when comparing the removal torque between the groups, but they followed the same pattern (TiT group > Ti group), demonstrating a relationship between these two measured parameters.

## 5. Conclusions

Within the limitations of the present animal study, the bone reaction (healing) around the titanium implants showed a more adequate interaction in comparison with the zirconia implants. In this way, the BIC% measured was very similar between the three groups; however, the torque removal values were superior for the titanium implants, which is related to faster bone mineralization on the titanium surface when compared to the zirconia surface for the proposed time period (6 weeks).

## Figures and Tables

**Table 1:** Mean of roughness values *S*_a_ and *R*_a_ (± standard deviation) of both groups.

Parameters	TiM Group	TiT Group	Zr Group
*S*_a_	0.18 (± 0.2)	0.77 (± 0.2)	0.17 (± 0.1)
*R*_a_	0.17 (± 0.1)	0.66 (± 0.3)	0.14 (± 0.2)
*Z*	0.92 (± 0.8)	2.61 (± 0.8)	0.77 (± 2.0)

*S*_a_ = average height of the analyzed area; *R*_a_ = arithmetic mean of absolute values of all profile points; *Z* = longest distance recorded among the peak and valley, high variation of the valleys.

**Table 2:** Comparison of the maximum removal torque (N cm) between the groups.

Group	Mean	SD	Median	Min	Max	*n*
TiM	15.9	4.18	16.1	8.4	22.1	10
TiT	27.9	5.15	27.9	15.1	35.3	10
Zr	11.5	2.92	11.1	7.3	16.0	10

SD, standard deviation. Min, minimum value. Max, maximum value. *n*, number of samples.

**Table 3:** Comparison of the bone-implant contact (%) between the groups.

Group	Mean	SD	Median	Min	Max	N
TiM	35.4	4.54	37.1	28.9	41.3	10
TiT	37.8	4.84	39.9	29.8	46.1	10
Zr	34.0	6.82	36.8	23.6	44.0	10

SD, standard deviation. Min, minimum value. Max, maximum value. *n*, number of samples.
